# Adult insect personality in the wild—*Calopteryx splendens* as a model for field studies

**DOI:** 10.1002/ece3.8439

**Published:** 2021-12-13

**Authors:** Maria J. Golab, Szymon Sniegula, Andrzej Antoł, Tomas Brodin

**Affiliations:** ^1^ Institute of Nature Conservation Polish Academy of Sciences Kraków Poland; ^2^ Department of Wildlife, Fish and Environmental Studies Swedish University of Agricultural Sciences Umeå Sweden

**Keywords:** *Calopteryx splendens*, field experiments, insect behavior, personality, repeatability

## Abstract

Animal personality has received increasing interest and acknowledgment within ecological research over the past two decades. However, some areas are still poorly studied and need to be developed. For instance, field studies focused on invertebrates are currently highly underrepresented in the literature. More studies including a wider variety of traits measured and species tested are needed to improve our understanding of trait‐correlation patterns and generalities. We studied nine behavioral traits, in the damselfly *Calopteryx splendens*, from an array of three experiments: (i) courtship, (ii) aggressiveness, and (iii) boldness, and calculated their repeatability. The behaviors were measured twice in two different contexts: (i) undisturbed territory and (ii) partially deteriorated territory. Traits related to courtship and boldness were all repeatable across the two contexts. Among aggressive behaviors, only one trait (number of hits) was repeatable. This work demonstrates, for the first time, the presence of within‐population personality differences in an adult damselfly in the wild. We further propose *C. splendens* as a promising model species for testing personality in the wild under highly controlled environmental conditions.

## Graphical abstract

*Calopteryx splendens* behaviors are highly repeatable. The presented field experiments allow to control for most environmental factors. Hence, the species and its habitat is a good model for personality studies in the wild.

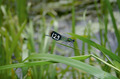

## 1 INTRODUCTION

Animal personality, defined as inter‐individual consistent differences in behavior across time and context, has received growing interest over the past two decades (e.g., Roche et al., 2016). Personality traits have important ecological and evolutionary implications for determining aspects such as space use, species geographic distributions, invasiveness, response to environmental change, speciation rates, social interactions, and fitness consequences (e.gBriffa & Weiss, 2010; Lichtenstein et al., 2017; Sih et al., 2004). One often used way to measure personality is repeatability, since repeatability is a highly informative metric providing a standardized estimate of consistency of individuality (Roche et al., 2016). Despite the obvious importance of animal personality, the research field is still facing some shortcomings. For example, there is a severe lack of field studies, in contrast to the vast number of laboratory‐based studies carried out (Archard & Braithwaite, 2010; Blaszczyk, 2020; Carere & Maestripieri, 2013; Frick et al., 2017; Hertel et al., 2020). This skew is unfortunate since laboratory‐based experiments often are affected by a number of constraints, for example, captivity stress, selective trapping, learning, homogeneity of the laboratory environment, artificial and relaxed selection, and reduced pool of potential mates (reviewed by Archard & Braithwaite, 2010). As a consequence, individuals in laboratory conditions may behave in ways not representative of the natural environment and hence showing ecologically irrelevant behavioral patterns (Niemelä & Dingemanse, 2014). Also, the amount of studies on invertebrate personality is drastically disproportionate to the number of species and behavioral traits (e.g., Kralj‐Fišer & Schuett, 2014). Further, most studies concern the “Big Five” of animal personality (boldness, aggressiveness, sociability, exploration, and activity), ignoring other traits that may bring in‐depth understanding of the phenomena and the possible associations between commonly and rarely measured traits (Koski, 2014). Finally, metrics of personality traits should be chosen with caution in order to be applicable for a given study organism and to represent ecologically relevant information (Carter et al., 2013).

Within‐species studies designed to compare laboratory‐based and field‐based assessments of personality show varying results; some fail to find any correlations between laboratory and field, whereas others provide similar personality estimates in the two environments. For example, a study on crickets (*Gryllus campestris*) made by Fisher et al. (2015) showed repeatability of exploration and activity both in laboratory and in natural conditions, but repeatability of shyness in artificial conditions only. Another study on zebra finches (*Taeniopygia guttata*) showed personality both in laboratory and in field conditions, but there was no correlation between behaviors under the two conditions (McCowan et al., 2015). In a recent example on sea anemones, Osborn and Briffa (2017) showed that the transition from field to laboratory environment might influence personality assessments. It is suggesting that the translocation itself can bias results (Niemelä & Dingemanse, 2017). On the contrary, studies on great tits (*Parus major*; Cole & Quinn, 2012) and striped mice (*Lemniscomys barbarus*; Yuen et al., 2016) showed that individuals behaved consistently both in the laboratory and in the field. This variation between laboratory and field results underlines the importance of increasing the number of field studies to further our understanding of the causes and consequences of animal personality in nature.

Despite the fact that invertebrates represent the most numerous group of animals on Earth (Larsen et al., 2017; Stork, 2018), personality studies on this taxa are still scarce compared with studies on vertebrates (Gosling, 2001; Kralj‐Fišer & Schuett, 2014; Mather & Logue, 2013). However, in recent years insects started to play an important role in animal behavioral research (Keiser et al., 2018). This is because insects display a wide range of sexual and social behaviors, many of which are rare or absent in vertebrates, providing new possibilities for addressing ecological or evolutionary questions connected to animal personality (Carere & Maestripieri, 2013). Other reasons to study insects are that they, often, are less ethically controversial and that studies covering the entire ontogeny, or several, are less time‐consuming because of a relatively short life span (Córdoba‐Aguilar et al., 2018). Despite these arguments, studies on insect personality in natural conditions are still rare (e.g., Fisher, James, et al., 2015).

Beyond the “Big Five” that became the blueprint for animal personality studies (Mather & Logue, 2013; Réale et al., 2007; Van Oers & Naguib, 2013), we have limited understanding of other behavioral traits. For example, behaviors related to mating have an extraordinary role in ecology and evolution, but have received relatively little attention in animal personality studies (Koski, 2014). For instance, the term “sociability” is used as a proxy for a whole range of behaviors. These include the following: hiding in the presence of a conspecifics' smell (Cote et al., 2008), grooming in chimpanzees (Koski, 2011), aggregation at food sources in fruit flies (Scott et al., 2018), tendency to shoal in mosquitofish (Brodin et al., 2019), and mating behaviors (Sih et al., 2014). It is possible that what researchers are calling sociability may actually represent different traits in different species (Koski, 2014). For instance, testing response to a predator (as a proxy of boldness) in open areas, which is used for, for example, kangaroos (Blumstein & Daniel, 2003), may not be adequate for a passerine, which usually inhabits and is preyed upon, in more closed habitats (Whittingham et al., 2004). One way to increase the accuracy of a personality measure is to carry out multiple tests of the personality trait (Carter et al., 2013), as was shown with guppies (*Poecilia reticulata*) where boldness was measured in three experiments (Burns, 2008).

Already‐established model organisms (i.e., non‐human species representing a larger group of organisms used for comparative and integrative research on specific scientific problems; Leonelli & Ankeny, 2013), intensively bred and studied under laboratory conditions over several decades, have their limitations and may not be very useful as models for some certain research. For instance, one of the most significant model organisms, the fruit fly (*Drosophila melanogaster*), intensively used for testing molecular mechanisms of behavior (Kain et al., 2012; Roberts, 2006; Sokolowski, 2001), has been reared for many generations in homogeneous environments of molecular biology laboratories. This has more than likely resulted in adaptation to the stable environment and, as a consequence, changing the behavioral reaction to novel conditions when compared to natural populations (Archard & Braithwaite, 2010). In a recent study on zebrafish, the behavior of wild animal was affected by exposure to anxiolytic pharmaceuticals, while the laboratory‐reared zebrafish was unaffected (Vossen et al., 2020). Hence, to increase the ecological relevance of studies including behavioral traits we need to both expand the number and taxonomic breadth of model organisms, and restock or replace existing laboratory populations (Behringer et al., 2009; Leonelli & Ankeny, 2013).

**FIGURE 1:**
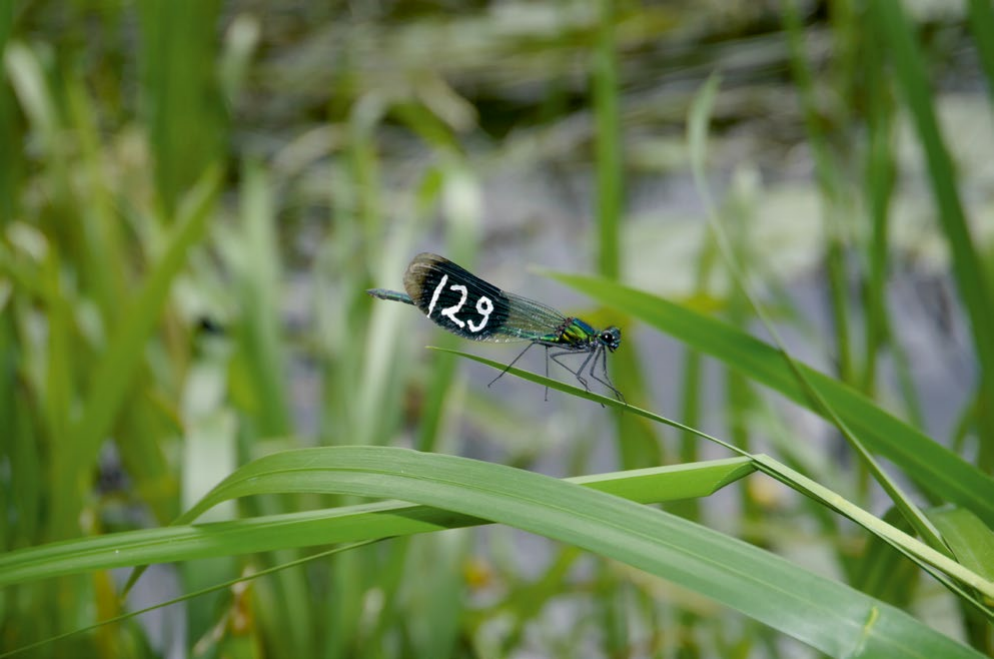
*Calopteryx splendens* male—study species

Here, we report repeatability of a set of behavioral traits over time and contexts in the damselfly *Calopteryx splendens* (Figure 1) measured in natural field conditions in order to discuss ecological relevance of personality studies under natural and laboratory conditions. We measured traits related to three behavioral axes: (i) courtship behavior, (ii) aggressiveness, and (iii) boldness. Since this is the first study on *C. splendens* personality in the wild, we test three traits within each behavioral axis to ensure their applicability to this study system (Carter et al., 2013). The repeatability was assessed in two different contexts: on undisturbed original patches (males' territories) and on partially deteriorated territories.

## 2 METHODS

### 2.1 Study species

Dragonflies and damselflies are considered as prime model systems for evolutionary and ecological research (Córdoba‐Aguilar, 2008). One of the most intensively studied families within Odonata is Calopterygidae (Córdoba‐Aguilar, 2008). *C. splendens* (Figure 1) is a very conspicuous representative from this damselfly family inhabiting lowland rivers in Europe (Askew, 1988; Dijkstra, 2006). *C. splendens* exhibits sexual dimorphism in body coloration, with blue reflecting dark wing spots in the middle of the wings of males, which is a trait easily recognizable from distance (Askew, 2004). Average life span of a mature male is ca. 1 week (Svensson et al., 2006; Svensson & Friberg, 2007; Tynkkynen et al., 2009), and the territorial and sexual behaviors of calopterygids, as well as traits determining *C. splendens* flight abilities, have been intensively studied over the past 20 years (Marden, 2008; Suhonen et al., 2008). *Calopteryx* sp. males defend territories (e.g., floating aquatic vegetation), with qualities (e.g., patch size, water current, plant composition) that correlate with a resident male success (e.gGibbons & Pain, 1992; Guillermo‐Ferreira & Del‐Claro, 2011; Plaistow, 1997; Siva‐Jothy et al., 1995). The species demonstrates a wide range of easily observed behaviors such as patrolling, aerial contests with conspecifics, and courtship dances (Golab et al., 2017; Marden & Waage, 1990; Rüppell et al., 2005; Waage, 1973). The flying patterns of different behaviors are very specific and easy to observe with the naked eye from a distance of several meters (Corbet, 1999; Golab et al., 2017; Pajunen, 1966). The species easily habituates to the observer, and after a disturbance in/of their environment, damselflies resume normal activity within minutes (Golab et al., 2013, 2017). The adult damselfly and its breeding sites are easily accessible to the investigator (Córdoba‐Aguilar & Cordero‐Rivera, 2005), and breeding site features can be manipulated or highly controlled (Golab et al., 2013, 2017). Trapping and behavioral observations do not affect individuals' behaviors (Golab et al., 2013, 2017), and methods of individual marking and field observations are well established and do not have overt effects on individuals (e.gGolab et al., 2017; Kuitunen et al., 2010; Plaistow & Siva‐Jothy, 1996). Adult *C. splendens* individuals have strong site fidelity, and less than 15% of population disperse more than 150 m (Schutte et al., 1997; Stettmer, 1996).

### 2.2 Study site and experimental setup

**FIGURE 2:**
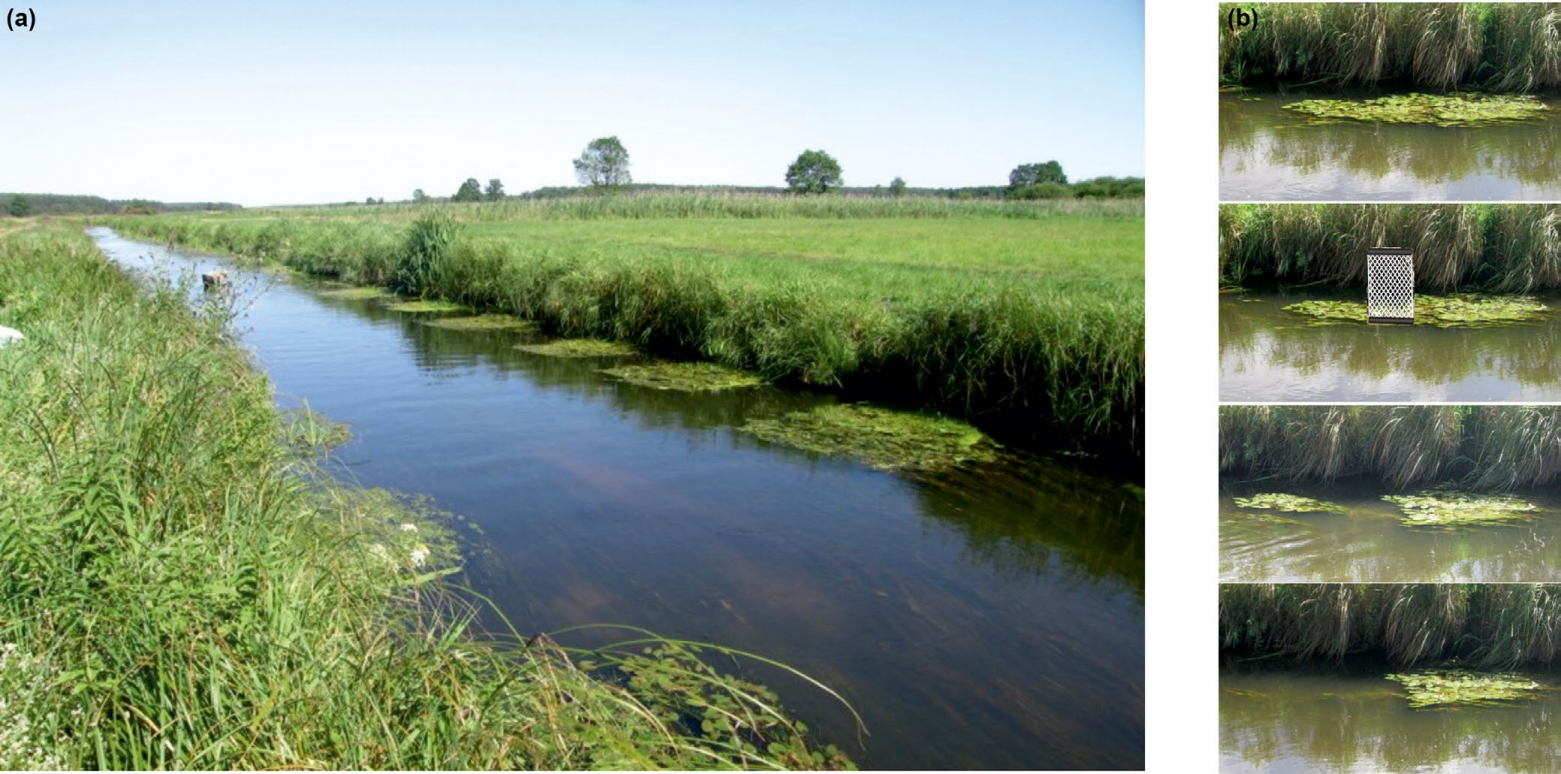
Study site in Biała Nida River including selected vegetation patches. Six patches of floating vegetation (*Potamogeton natans*) manipulated to equal size, used as territories by males and oviposition substrate by females (a). Sequential experimental patch area reduction using a ballast (b)

Experiments were conducted between June 15 and July 31, 2015 and 2019, on a 50‐m section of River Biała Nida (Figure 2), in southern Poland. To reduce the possible influence of weather on damselfly behaviors, the studies were performed during warm, dry, and low‐wind weather (Golab et al., 2013; Tynkkynen et al., 2004). Both the riparian and the floating vegetation were cut with a pair of scissors so that the composition and the spatial structure were homogeneous. The size of all floating vegetation rafts (patches) that are defended by males as their territories and used by females as oviposition sites was similar/comparable among patches, and the dimensions were ca. 2.5 × 2 m (Golab et al., 2013, Figure 2). These conditions minimized any microclimate differences between the studied territories. Also, predation during the experiments was controlled/limited to a minimum. The only predators of *C. splendens* at the study river section that could possibly affect the experiments (e.g., hunt studied territorial males) were birds, and these did not enter the study area due to the presence of 2–3 observers. Other possible predators were not recorded at the study area (Golab and Sniegula, personal observation).

Data collections were run between 1000 and 1600 h CEST. First, all *C. splendens* individuals present at the studied section of the river were caught with entomological hand net and individually marked with a three‐digit numbers (white marking pen). Then, five randomly chosen mature males and five females were caught and glued to the fishing line (Tynkkynen et al., 2008, Figure 3), placed in a cool box to prevent energy expenditure, and stored until experiment begins. The method has been previously tested to eliminate the risk of adverse effects on both flying ability of presented individuals and response of tested (focal) males. Experimental bouts were preceded by a 10‐min observation of studied territories (patches) in order to assign resident males to their territories. The age of males was assigned to 4 age categories (1—immature; 2—mature with soft wings; 3—mature without visible wing wear; and 4—mature with some wing damages; Golab, personal observation). Only males from category 3 were chosen for further studies, since the age can influence male territorial behavior (Corbet, 1999; Tynkkynen et al., 2009) and could influence a males' response to our experimental treatments.

**FIGURE 3:**
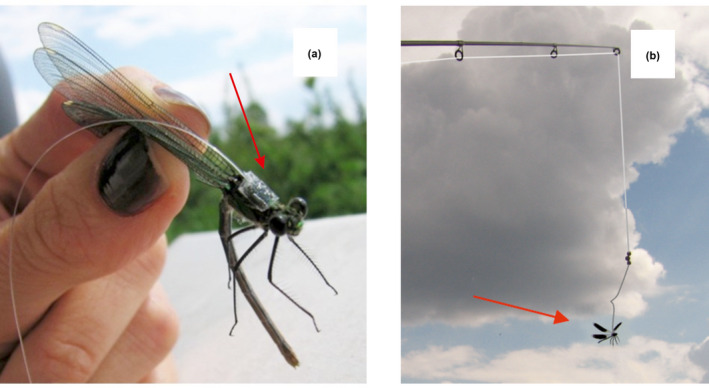
Experiment in the field. (a) *C. splendens* female glued to a fishing line; (b) flying *C. splendens* male glued to a fishing line

For each resident male, three types of experiments were run: (1) courtship experiment—female attached to a fishing line was presented to a focal resident male for 2 min; (2) aggressiveness—a male attached to a fishing line was presented to a resident male for 2 min; and (3) boldness—a bird decoy was moved toward a resident male (simulated predator attack) until he flew away and latency to return to his territory was measured (Table 1). In every experiment, we measured three traits (described in Table 1). Each experiment was run twice: on the original patches (morning trial) and repeated on the patches manipulated by sinking ca. 25% of each vegetation patch using a ballast (afternoon trial) (Figure 2b). The minimum time between the two rounds of the experiment was 1 h. Males and females on the fishing line were replaced by new ones every 20 min, in order to avoid exhaustion or rejection signals in case of females (Tynkkynen et al., 2008). The patch manipulation aimed at measuring the traits across two situations and times (Dingemanse & Reale, 2005; Sih et al., 2004).

**TABLE 1:** Behavioral traits measured in three experiments on *C. splendens* in Biała Nida River

Experiment	Traits	Description
Courtship	Reaction to female	Time [s] until focal resident moved toward a female
	Dive display	Number of alighting of a resident male on the water surface (a common courtship display in *Calopteryx* sp.)
	Engagement	A nominal value describing a male devotion to courtship: 100%: male attempts to form a tandem, dives on water, patrols a territory, chases away intruders, does not fly aside (does not leave his territory during the experimental period), does not perch (does not stop flying during the experiment); 75%: male patrols a territory, chases away intruders, does not fly aside, does not perch 50%: male patrols territory, chases away intruders, flies aside, perches; 25%: no patrolling, no chasing, flights aside, perching during the experimental trial
Aggressiveness	Reaction to intruder	Time [s] until focal resident moved toward the presented intruder male
	Bites	How many times a resident male bit the intruder
	Hits	How many times a resident male hit the intruder
Boldness	Distance to react	Binomial distance value: Near—male escaped when the bird decoy was closer than 0.5 m Far—male escaped when the bird decoy was more than 0.5 m away
	Escape distance	Distance [m; with an accuracy of 0.5 m] the focal resident male flew after the predatory attack simulation
	Time to return	Time [s] passed until the resident returned to his territory after the predatory attack. The maximum time the observer waited for the male to come back was 180 s

### 2.3 Statistical analyses

We assessed behavioral consistency (i.e., personality) by quantifying repeatability coefficient for the nine traits using the package “rptR” in R v.4.0.2. (R Development Core Team, 2016; Stoffel et al., 2017). Repeatability coefficient (*R*) calculated as ratio of group‐level variance over the sum of group level and residual variance gives the information about how particular trait correlates between replication in one replication unit (in our case, individual). The coefficient takes value between 0 and 1. In rpt models, we set the bootstrap number to 1000 in order to properly estimate confidence intervals for repeatability coefficient.

## 3 RESULTS

The estimated population density, assessed based on a standardized daily mark–release procedure, was ca. 1 individual per 1‐m section of the river, which is an intermediate density for the species (Chaput‐Bardy et al., 2010; Kuitunen et al., 2010; Stettmer, 1996).

All behavioral traits related to courtship and boldness were significantly repeatable. In contrast, among the traits connected to aggression only “number of hits” was repeatable (Table 2).

**TABLE 2:** Repeatability (R) of behavioral traits of *C. splendens* in Biała Nida River

Trait	R	*p*	95% CI
Reaction to female	0.396	**<.001**	0.174, 0.553
Dive display	0.338	**<.001**	0.184, 0.465
Engagement	0.492	**<.001**	0.339, 0.624
Reaction to intruder	0.167	.055	0, 0.332
Bites	0.160	.109	0, 0.347
Hits	0.364	**<.001**	0.113, 0.541
Distance to react	0.396	**<.001**	0.174, 0.553
Escape distance	0.338	**<.001**	0.161, 0.491
Time to return	0.282	.**001**	0.098, 0.440

Note

Bold values indicate statistical significance of *p*‐values.

## 4 DISCUSSION

We report for the first time personality in a natural population of the damselfly *C. splendens* measured in the wild. We show cross‐context repeatability in most of the traits studied. Traits related to both courtship and boldness axes showed repeatability values close to the average value of behavioral repeatability across over 750 studies of various behavioral traits and taxa (Bell et al., 2009). Our research responds to the need to study personality under natural field conditions in order to assess ecologically relevant situations and contexts (e.gArchard & Braithwaite, 2010; Hertel et al., 2020). Further, our study indicates that *C. splendens* is a suitable candidate as a model organism in behavioral studies.

The number of hits seemed to be a good proxy of aggression in *C. splendens* in the wild. Generally, *Calopteryx* spp. males compete for resources during aerial contests (Marden & Waage, 1990), but most of the disputes are brief pursuit flights after which an intruder is chased away. During longer aerial fights, collisions or hitting can occur (Rüppell et al., 2005; Golab, personal observation) and the intensity likely depends on the combination of male personalities interacting. Generally, individuals' latency to approach a rival and number of bites are two of the most commonly used indicators of aggressiveness and show high repeatability in most studies (Keiser et al., 2018). In our case, the absence or low repeatability for both reaction to intruder and number of bites (Table 2) is in contrast to that trend and is also in contrast to an earlier meta‐analysis (Bell et al., 2009). However, in crickets, Fitzsimmons and Bertram (2013) showed low repeatability of aggression scores (quantified from the duration and frequency of agonistic behaviors during contest). These authors suggested that the trait plasticity was an effect of the social environment and physiology (Fitzsimmons & Bertram, 2013). In our study, the intruder male was chosen randomly, and we did not measure their physiological condition, which might have been useful for a deeper understanding of our result. Also, we propose that future studies should use a mirror (e.g., Balzarini et al., 2014) instead of an actual rival, which will generate more controlled and comparable metrics of aggression. Based on our results, we suggest that biting an intruder is a plastic trait that depends on weather, position of the rivals, and intrusion time (Rüppell & Hilfert‐Rüppell, 2013). Hence, it is unsuitable for personality measures in the studied species. In aerial contest (Marden & Waage, 1990), biting the intruder may arise by chance, depending on the direction/intensity/frequency of the damselfly movements. Also, some parts of the body may simply be harder to bite, for instance the center of a wing area. Additionally, despite the fact that dragonflies have a very advanced visual system (Bybee et al., 2012) and can compute flight trajectory of their prey (Olberg, 2012), there is no evidence that odonates would be able to compute their opponents' movements and precisely bite one another.

The time needed for reaction to rival male showed no consistency in our study (Table 2). We suppose the trait might be strongly influenced by the social environment and hence more plastic (Bell et al., 2009). In this species, antagonistic behaviors depend on whether the potential rival male is a neighbor, a wandering male, or an actual opponent. It has been shown that neighboring territorial males avoid contest (Briffa & Weiss, 2010; Golab et al., 2017; Gordon, 1997; Maynard Smith & Parker, 1976). Also, non‐territorial *C. splendens* males often patrol larger sections of a river looking for territories or mates. During this activity, non‐territorial males may either pass a given territory, approach the resident and retreat immediately, or initiate a conflict of varying intensity (Koskimäki et al., 2009; Panov & Opaev, 2013). Resident males must evaluate which of the three types of males he is facing and react adequately to the situation. The above discussed results illustrate the importance of choosing the right test for estimating a personality trait (Sih & Bell, 2008) and that developing multiple proxies for a given behavioral axis might be crucial to identify the most suitable test for the targeted trait in a given species (Carter et al., 2013).

The time a resident male needed to react to an approaching female showed individual consistency (Table 2). In odonates, a resident male usually wants to attract a female rather than to chase her away (Corbet, 1999). In addition, the other two traits related to courtship: Dive display and engagement were also consistent and, as such, potentially good metrics for personality. Consistent engagement to mating displays has also been shown, for instance, in male guppies (Biro et al., 2016; Magellan & Magurran, 2007). This is all in accordance with theory that predicts consistency in mating behaviors since it reduces cognitive costs for potential mates (Dall et al., 2004). Also, females can select a sexual partner of a behavioral profile adaptive for her offspring in a given environment (Réale et al., 2007). On the contrary, highly variable or unpredictable environments would favor behavioral plasticity rather than consistency (Dingemanse et al., 2010). Hence, there are studies, in opposition to our results, showing no personality in mating‐related behaviors, for example, in subdominant reindeers, whose propensity to enter/visit mating group is based on proximate factors such as the group sex ratio and a day of mating season (Strong, 2015).

Among insects, one of the most advanced personality research in the wild has been conducted on crickets. In a large‐scale project “Wild Crickets” (https://www.wildcrickets.org/), a group of researchers studied personality both in the field and in the laboratory. They found that individual behavioral consistency is stable over adult lifetimes (Fisher et al., 2015) and that personality in captivity not always predicts personality in nature (Fisher, James, et al., 2015). This is in line with another study on crickets, *G. campestris*, showing that handling procedure in translocation experiments may bias repeatability estimates (Niemelä & Dingemanse, 2017). In our study, we did not compare field with laboratory‐based experiments. However, since there is growing evidence that gene expression can be significantly modified by environmental factors (Niemelä & Dingemanse, 2014) and artificial conditions can impose additional unnatural stressors (Archard & Braithwaite, 2010), we conclude that, for most animals including adult calopterygid damselflies, field studies are superior, in ecological relevance, compared with laboratory‐based experiments. More specifically, the methods presented in this study are particularly promising for studying adult damselfly behavior since they reduced handling trauma, did not influence natural/free behavior of damselflies during the observations (Golab, personal observation), and prevented damselflies from adjusting/habituating to the procedure (Archard & Braithwaite, 2010; Hilfert‐Rüppell, 1999).

One common difficulty when studying animal personality in the field is controlling environmental heterogeneity (Bell, 2004; Dingemanse & Réale, 2013; Quinn et al., 2009). In our experiments, many environmental factors such as comparable microclimate (sunlight penetration, air temperature, wind speed), water quality (current, temperature, velocity), and composition and structure of macrophytes and predation were standardized (Golab and Sniegula, personal observation, details in methods). This adds a robustness to our results that often can be lacking in animal personality field studies due to varying environmental conditions.

We want to emphasize that *Calopteryx* spp. express other ecologically important behaviors, beyond the “Big Five”(Keiser et al., 2018), that could be potentially useful for future personality studies (Koski, 2014). These traits include the following: territory patrolling (Corbet, 2004; Golab et al., 2017), gathering of non‐territorial males (Golab et al., 2013), and a very elaborated repertoire of courtship behaviors (Corbet, 2004; Rüppell et al., 2005). To summarize, our work is the first that demonstrate behavioral repeatability in an adult damselfly in the wild. Our results suggest that adult *C. splendens* is a very promising model organism for studying insect personality under ecologically relevant natural conditions. The species has an elaborate repertoire of behaviors that can be easily observed and measured swiftly using only the naked eye. In addition, they also have a strong site fidelity, which enables controlled and relevant manipulations of key environmental parameters. We suggest that our study represents the natural variability that exists in studied behaviors of this species. Two of the traits related to aggressiveness were not consistent and are unlikely to be useful for personality tests or experiments. This emphasizes the need of proper trait selection when aiming to understand ecological implications of differences in individual behavior. Also, further studies on different behaviors of *C. splendens* in various contexts and situations may be highly relevant for understanding the ecological and evolutionary causes and consequences of animal personality (Wolf & Weissing, 2012) in natural populations (Archard & Braithwaite, 2010; Osborn & Briffa, 2017).

## CONFLICT OF INTEREST

None declared.

## AUTHOR CONTRIBUTIONS

**Maria J. Golab:** Conceptualization (lead); data curation (equal); formal analysis (supporting); funding acquisition (lead); investigation (lead); methodology (lead); project administration (lead); resources (lead); supervision (equal); validation (lead); visualization (lead); writing – original draft (lead); writing – review & editing (equal). **Szymon Sniegula:** Conceptualization (supporting); investigation (equal); methodology (supporting); validation (supporting); writing – original draft (supporting); writing – review & editing (equal). **Andrzej Antoł:** Data curation (supporting); formal analysis (lead); methodology (supporting); software (supporting); writing – review & editing (supporting). **Tomas Brodin:** Conceptualization (supporting); funding acquisition (supporting); methodology (supporting); supervision (equal); validation (supporting); writing – original draft (supporting); writing – review & editing (equal).

## Data Availability

Data are openly available in Dryad at https://doi.org/10.5061/dryad.kd51c5b72.
